# SILVI, an open-source pipeline for T-cell epitope selection

**DOI:** 10.1371/journal.pone.0273494

**Published:** 2022-09-07

**Authors:** Joana Pissarra, Franck Dorkeld, Etienne Loire, Vincent Bonhomme, Denis Sereno, Jean-Loup Lemesre, Philippe Holzmuller

**Affiliations:** 1 UMR INTERTRYP, IRD, CIRAD, University of Montpellier (I-MUSE), Montpellier, France; 2 UMR CBGP, INRAE, CIRAD, IRD, Montpellier SupAgro, University of Montpellier (I-MUSE), Montpellier, France; 3 UMR ASTRE, CIRAD, INRAE, University of Montpellier (I-MUSE), Montpellier, France; 4 ISEM, CNRS, EPHE, IRD, University of Montpellier (I-MUSE), Montpellier, France; National Cancer Institute at Frederick, UNITED STATES

## Abstract

High-throughput screening of available genomic data and identification of potential antigenic candidates have promoted the development of epitope-based vaccines and therapeutics. Several immunoinformatic tools are available to predict potential epitopes and other immunogenicity-related features, yet it is still challenging and time-consuming to compare and integrate results from different algorithms. We developed the R script SILVI (short for: from *in silico* to *in vivo*), to assist in the selection of the potentially most immunogenic T-cell epitopes from Human Leukocyte Antigen (HLA)-binding prediction data. SILVI merges and compares data from available HLA-binding prediction servers, and integrates additional relevant information of predicted epitopes, namely BLASTp alignments with host proteins and physical-chemical properties. The two default criteria applied by SILVI and additional filtering allow the fast selection of the most conserved, promiscuous, strong binding T-cell epitopes. Users may adapt the script at their discretion as it is written in open-source R language. To demonstrate the workflow and present selection options, SILVI was used to integrate HLA-binding prediction results of three example proteins, from viral, bacterial and parasitic microorganisms, containing validated epitopes included in the Immune Epitope Database (IEDB), plus the Human Papillomavirus (HPV) proteome. Applying different filters on predicted IC50, hydrophobicity and mismatches with host proteins allows to significantly reduce the epitope lists with favourable sensitivity and specificity to select immunogenic epitopes. We contemplate SILVI will assist T-cell epitope selections and can be continuously refined in a community-driven manner, helping the improvement and design of peptide-based vaccines or immunotherapies. SILVI development version is available at: github.com/JoanaPissarra/SILVI2020 and https://doi.org/10.5281/zenodo.6865909.

## Introduction

In the post-genomic area, available databases and -omics datasets have been extensively explored to discover antigens for the development of vaccines or immunotherapies [1]. Reverse vaccinology (RV) approaches hold the promise for breakthrough contributions to vaccine development, as in the case of the meningococcal vaccine [2]. Yet, there are still a myriad of pathogens and conditions for which no vaccine has yet been successfully developed, particularly in the case of Neglected Infectious Diseases (NIDs), wherein current tools, innovation and funding are lacking, and would greatly benefit of new preventive or therapeutic candidates with low development costs [3, 4].

To induce protective immunity, epitope-based vaccines require identifying the minimal immunogenic units for optimal recognition by the host’s immune system and induction of protective immunity [5, 6]. Also, epitope-based selections offer great advantages when compared to empirical antigen selection, and can be applied whenever cellular immune responses are relevant, whether protective or detrimental [7–10]. Yet, epitope selection from databases remains a challenging step to optimise and diversify the antigens to be tested in an experimental setting [10].

Epitope immunogenicity relies firstly on high-affinity binding to HLA-class I or -class II molecules and on antigen abundance and kinetics of expression, conservation, low homology to the host, intracellular processing, and the presence of T cells with specific TCRs [11–13]. To inform on these features, databases such as Immune Epitope DataBase (IEDB) and other tools are available [14]. Nevertheless, *in silico*-based approaches have not supported a significant increase in the total number of licensed products in the last decades and several challenges remain [9, 15, 16]. Our capacity to explore the vast amount of data generated by immunoinformatic algorithms is still limited to a few often web-based tools that work with different output formats. Currently available web-servers allowing RV pipelines are: the New Enhanced Reverse Vaccinology Environment (NERVE), Vaxign, or The Jenner-Predict [17–19]. They provide insights on the immunogenic potential of protein antigens and reduce the initial protein candidates to test. However, these pipelines present some setbacks, such as the limited number of input sequences and limited number of available genomes. Also, some apply automatic cytosolic/surface expression filters, which are not transversal to all pathogens or diseases (i.e. in the case of exosome-based secretion). Therefore, they may overlook key targets [20] and restrict options and selection parameters.

Optimised T-cell epitope selection relies on knowledge of several disease-specific variables: host susceptibility/resistance factors, HLA variability, environmental factors [21, 22] and pathogen-related characteristics such as virulence, tropism, immunomodulation and species conservation [23].

Several open-access immunoinformatics tools and databases are available to evaluate a set of characteristics associated with immunogenic epitopes [6, 24–26]. T-cell epitope prediction can be performed via direct prediction (predicting T-cell receptor, TCR recognition) or indirect methods (predicting epitope binding to HLA molecules), the latter extensively more accurate than the former [27]. HLA-binding affinity has become the first criterion when trying to predict if a given peptide sequence constitutes an epitope, since it is the first requirement for T-cell activation and it correlates with peptide linear sequences [26, 27]. Machine-learning algorithms, such as Artificial Neural Networks (ANNs) and Support Vector Machines (SVMs), display a good predictive performance for HLA-binding predictions [28–31]. HLA-class II binding predictions are currently slightly less accurate than HLA-class I binding predictions because they involve conformational criteria [32, 33]. Besides, HLA-class II epitopes are longer (around 15 to 25-mer) and several binding registers or cores may be present in the same peptide [34, 35].

Additional criteria for *in silico* predictions of epitope immunogenicity include: i) protein abundance, subcellular localization and expression dynamics, as abundant and early expressed pathogen-specific epitopes have increased chances of being processed and presented [36, 37]; ii) peptide-MHC complex (pMHC) binding affinity and stability [12, 38, 39]; iii) efficiency of pMHC processing [24]; iv) homology, either as positive selection criterion of conserved sequences among pathogenic species, or as negative selection criterion of sequences homologous to host proteins [27]; v) other biochemical properties such as solubility help guide peptide selection, formulation and handling [12, 28].

The selection steps involving analysis of subcellular localization, abundance and good expression dynamics are the filters with the highest selective power [36]. Completely conserved epitopes are ideal for pan-vaccine development across multiple pathogenic strains [23]. Furthermore, a combined approach that uses numerous predictors will increase the confidence in the predicted peptides’ binding affinity, restriction and immunogenicity [35, 40]. Criteria that can be interesting for peptide selection are proteasomal processing predictors, however, there are still significant knowledge gaps and no significant evidence of a good selective power, making them low prediction efficiency algorithms [36, 41]. The criteria of high peptide homology to host proteins can be considered as an unreliable filter since self-recognition depends on the TCR-pMHC interaction which allows a reasonable amount of molecular mimicry, and is therefore difficult to predict [42–44]. Nevertheless, potential interferences resulting in autoimmunity are correlated with epitope conservancy. So, BLASTp alignments can be used to compare pathogen- and host-derived peptide sequences, to describe similarities through position-specific mismatches, a feature which also included in the NERVE pipeline. Considering all this, we propose that a robust epitope selection process should start with binding affinity prediction analysis by at least two different algorithms, of a strong antigen pool (highly abundant, conserved, exposed and accessible proteins, expressed in the appropriate timing during infection). Additional adjustable filters are homology to host proteins, promiscuity, binding affinity, and solubility, with which we can rank epitopes.

Here we developed a workflow for epitope selection under R, named SILVI (short for: from *in silico* to *in vivo*). The script reads epitope binding prediction data from different predictors, processes and compares data, assimilates BLASTp alignment results [45] and feeds a final output table with all relevant information to perform the tailored epitope selections with available information, thus helping to refine the search of the most immunogenic epitopes. We demonstrated the relevance of SILVI’s workflow with epitope selection from the Genome Polyprotein (P26664 and P27958) from Hepatitis C Virus (HCV), the Circumsporozoite Protein (P19597) from *Plasmodium falciparum*, the 6kDa early secretory antigenic target (P9WNK7) from *Mycobacterium tuberculosis*, and the Human Papilloma Virus (HPV) proteome (uniprot_HPV_proteome_UP000126093). SILVI helps the process of epitope selection from a vast amount of data produced by different open-source third-party algorithms, and to add extra relevant information, in a non-restrictive, user-friendly manner. SILVI is readily available for use and due to the versatility and open-access nature of the R language, it can be improved, expanded, and easily tailored to meet users’ specific research needs.

## Results

### T-cell epitope selection with SILVI on example proteins from four pathogenic microorganisms

The four examples used to demonstrate SILVI’s workflow include one viral protein with two strain-specific sequences (HCV Genome Polyprotein, P26664 and P27958); one bacterial protein including one sequence from *M*. *tuberculosis* (P9WNK7); one parasitic protein including one sequence from *P*. *falciparum* isolate NF54 which contains several repeated 9-mers (P19597); and, finally, the full proteome of HPV (uniprot_HPV_proteome_UP000126093, taxon identifier 10566) which encodes 6 proteins (Table 1).

**Table 1:** Example proteins and validated epitopes present in the IEDB 3.0 database.

	example#1 HCV GP	example#2 Pf CSP	example#3 Mtb EsxA	example#4 HPV proteome
**Organism**	Hepatitis C virus	*Plasmodium falciparum*	*Mycobacterium tuberculosis*	Human papilloma virus
**Protein name**	Genome Polyprotein	Circumsporozoite protein (CSP)	6 KDa early secretory antigenic target (EsxA)	Complete proteome (HPVproteome)
**UniprotKB accession**	P26664 from HCV genotype 1a (isolate 1) and P27958 from HCV genotype 1a (isolate H77)	P19597 from *P*. *falciparum* (isolate NF54)	P9WNK7 from *M*. *tuberculosis* (strain ATCC 25618 / H37Rv)	uniprot_HPV_proteome_UP000126093 from HPV (taxon identifier 10566)
**length (a.a.)**	3011	397	95	2309 (6 proteins with 400/139/ 96/600/555/519 a.a.)
**validated 9-mer epitopes** ^1^	106	24	42	None
**validated 15-mer epitopes** ^1^	122	27	59	None

^1^retrieved from IEDB.org.

**Fig 1:**
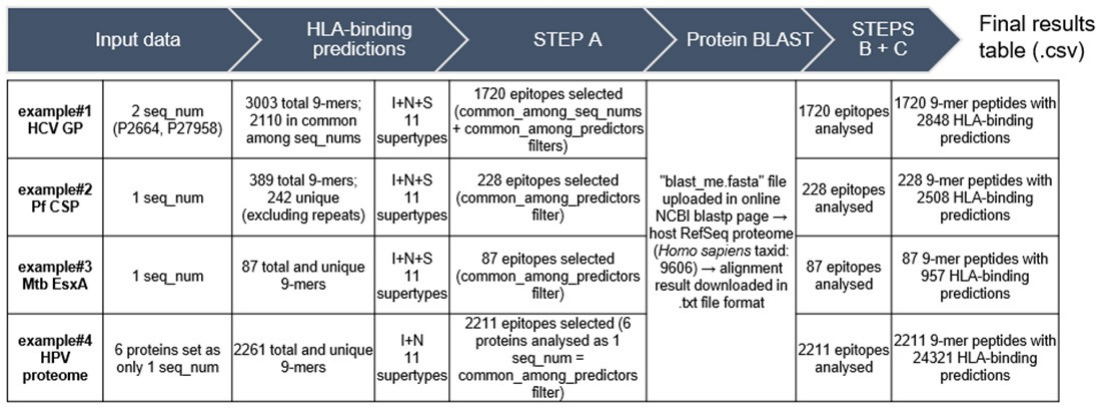
Results for HLA-class I T-cell epitope selection with SILVI on the 4 example proteins. I, IEDB MHC-I binding (consensus); N, NetMHCpan; S, SYFPEITHI.

**Fig 2:**
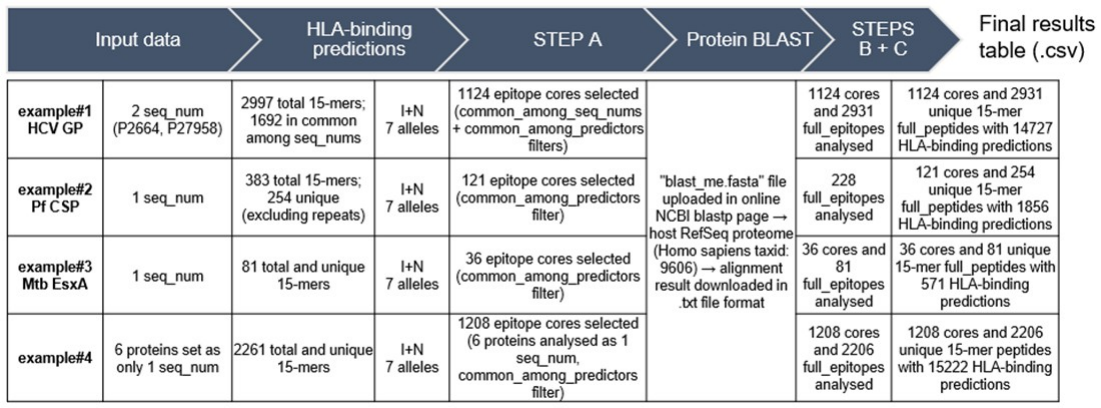
Results for HLA-class II T-cell epitope selection with SILVI on the 4 example proteins. I, IEDB MHC-II binding (consensus) algorithm; N, NetMHCIIpan.

Individual protein fasta files, containing one or more sequences, corresponding to the selected examples were directly uploaded in the algorithms’ web-servers and HLA-class I predictions were performed for all total and unique 9-mer peptides (Fig 1). HLA-class II predictions were performed for all 15-mer full peptides (Fig 2).

With SILVI’s step A applied to the example proteins, the peptides shared between species-specific protein sequences (*seq*_*nums*) are selected (*common_among_seq_nums* filter), and only the epitopes predicted by at least two predictors are selected (*common_among_predictors* filter). In the examples, proteins were analysed with only one *seq_num* (Pf CSP, Mtb EsxA and HPV proteome), or two *seq_nums* (HCV genome polyprotein). SILVI integrated data from all 9-mer or 15-mer epitopes and respective HLA-binding prediction information, meaning only repeated epitopes (illustrated by Pf CSP) or epitopes not shared by species-specific sequences (illustrated by HCV genome polyprotein) were excluded from the analysis.

In the BLASTp online server, the human RefSeq proteins were used as the host proteome (*Homo sapiens*, Taxid: 9606). The corresponding.txt result files were downloaded to the working directory and SILVI’s steps B and C were run sequentially (Figs 1 and 2). The intermediary output files “2_common_blast.csv” and “3_blast_mismatches.csv”, include the short-blastp results, position-specific mismatches, and physical-chemical properties (package Peptides) in addition to HLA prediction data. Step C generated the final result files (res_classI.csv and res_classII.csv), in which the IC50 value predicted by NetMHCpan (“scoreN”) for a given peptide or full_peptide/core combination and final match/mismatch counts are added.

Since no filters were applied (e.g. percentile rank or IC50) on initial data, final binding predictions include strong-, low- and non-binding 9-mer and 15-mer peptides. For HLA-class I binding prediction data, and for the examples with only one *seq_num* (Pf CSP, Mtb EsxA and HPV proteome), all 9-mer epitopes are analysed in the final output table and promiscuity is always equal to 11 (total number of supertypes considered by default). Similarly, for HLA-class II results, all epitope cores are analysed in the final output table. However, because both algorithms may not predict a given full_peptide/core combination, it will not be assigned HLA restriction (“NA”) or IC50 value (“scoreN = NA”).

### Comparison of SILVI’s outputs with IEDB-validated epitopes

Previously validated epitopes specific to the selected example proteins were retrieved from the IEDB 3.0 database to exemplify SILVI’s workflow. From full epitope lists from the example proteins, all 9-mer and 15-mer validated epitopes were retrieved (S1 Table). Information from the final output files was used to characterize the validated epitopes in the IEDB database (S1 Table) and possibly hint at the most relevant filtering criteria users can utilize to reduce epitope lists (S1 and S2 Figs).

For HCV GP (example#1), from the total 3003 9-mer peptides present in both protein sequences, 2109 are shared among the two *seq_nums* P26664 and P27958. Similarly, among the 2997 15-mer peptides present, 1692 are shared among the two *seq_nums*. The total 2519 epitopes in IEDB for the HCV GP, 256 are 9-mer linear peptides and 324 are 15-mer linear epitopes (23%). Some of these validated epitopes are not present in the selected sequences (150 9-mers and 202 15-mers), for a total of 106 9-mer and 122 15-mer validated epitopes present in P26664 and P27958 which were characterized with data from SILVI’s final output table (S1 and S2 Figs).

For Pf CSP (example#2), 294 epitopes are present in IEDB from several CSP and CSP-related antigens from different *P*. *falciparum* isolates, including 41 9-mer and 30 15-mer validated epitopes (24%). Among these, 36 9-mer epitopes are from the CSP antigen (excluding related proteins), 24 of which are present in the selected P19597 protein (S1 Fig). Similarly, CSP has 29 15-mer epitopes in IEDB, 27 of which are present in the P19597 protein (S2 Fig).

Interestingly, the strongest binding validated HLA-class I epitopes from the HCV GP and Pf CSP proteins have higher overall predicted binding affinity (IC50 value calculated by NetMHCpan, “scoreN”), with over 75% and 62% of validated epitopes under 1100 nM predicted IC50, respectively, whereas for Mtb EsxA, only 19% of validated epitopes are below this cutoff (S1 Fig). The overall average of best IC50 predictions for validated epitopes is 1618.81 nM (±3220.9) for HCV GP, 2439.32 nM (±4323.43) for Pf CSP, and 8177.56 nM (±7677.85) for Mtb EsxA.

Concerning overall HLA-class I predictions, the presence of two *seq_nums* (HCV GP) greatly restricted the final epitope list and predictions, and 94% of the total 256 predictions on the 102 validated epitopes in the final output table are below 1100 nM predicted IC50 (S1 Fig). Pf CSP and Mtb EsxA, with only one *seq_num* and no binding affinity filter applied to initial HLA-binding predictions, include only 10% and 2% total predictions below the same cutoff, respectively (S1 Fig).

Most HCV GP and Mtb EsxA 9-mer epitopes present hydrophobicity values between -1 and 1 (62% and 88%, respectively), whereas only 37,5% of Pf CSP peptides are within this range. Most allele supertypes are represented in the examples with a superior representation of the A02 (17–28%), B44 (13–17%) and B62 (10–26%) supertypes (S1 Fig). Regarding total mismatches against human proteins, an overwhelming majority of epitopes of all examples possesses between 1 to 3 mismatches (72–94%), and roughly a quarter (24–29%) of all 9-mer epitopes have 0 anchor position mismatches, while 55–97% have at least 1 anchor position mismatch (S1 Fig).

Regarding the best predicted affinity for validated 15-mer epitopes, HCV GP has the highest number of validated epitopes predicted to bind with an IC50 below 1100 nM (44%), while for Pf CSP and Mtb EsxA only 34% and 16%, respectively, are under this cutoff value (S2 Fig). Overall average IC50 for the best predictions of validated 15-mer epitopes is 1580.76 nM (±2803.11) for HCV GP, 1530.05 nM (±1769.1) for Pf CSP, and 2214.4 nM (±2201.65) for Mtb EsxA.

At least 48% of validated Pf CSP 15-mer epitopes present a hydrophobicity value between -1 and 1, increasing to 72 and 92% of validated epitopes from HCV GP and Mtb EsxA, respectively. All Mtb EsxA validated 15-mer epitopes present 1 to 3 total mismatches with human proteins, decreasing to 79% for HCV GP, and 64% for Pf CSP (S2 Fig).

Among the best HLA-class II predictions for validated epitopes, and for all example antigens, most predict binding to HLA-DRB1*07:01 (38–49%) and HLA-DRB4*01:01 (7–22%) (S2 Fig). Epitope promiscuity, for HLA-class II predictions, pertains to binding core predictions by the different algorithms, and in case of disagreement between predictors, promiscuity equals zero. Results show the nearly 100% of validated epitopes present a promiscuity ranging from 1 to 6 alleles, comprising 46–52% of epitopes predicted to bind to 5 or 6 alleles (S2 Fig).

#### Validated HLA-class I epitopes in final output table (“res_classI.csv”)

For HCV GP, the highest predicted IC50 corresponds to peptide ADTAACGDI, 18184.5 nM binding affinity to alleles from the B44 supertype. This epitope in IEDB (ID 775) has 2/2 positive experimental results but for binding to mouse H2-Kk alleles (Mamu-A1*011:01 and H2-Kk) (S1 Table). For epitope ID 6435 (CINGVCWTV), the best predicted binding affinity is specific to alleles belonging to the A02 supertype (321 nM), which agrees with the experimental data regarding the HLA restriction of this epitope in the IEDB database (303 positive assays) (S1 Table) [46, 47]. This peptide is also predicted to bind with extremely low affinity to the alleles from the A01 supertype (21497.1 nM) by all predictors. Experimental data is extensive for this 9-mer peptide, with 342 assays in total and only 30 with negative results (<9%), including positive results for binding to HLA-A3 alleles (chromium-51 cytotoxicity) and HLA-class II molecules (ELISA IL-2 release) (S1 Table). From the 102 validated 9-mer peptides present in the final output table for HCV GP, only 9 were not assigned the same HLA restriction by at least two predictors (promiscuity = 0). As an example, HCV GP epitopes EVVTSTWVL (ID 14902) and DVVCCSMSY (ID 10763) are predicted to bind strongly to alleles from the A26 supertype (187 and 37 nM, respectively) which correlates with experimental data in IEDB (3 positive assays out of 7, and 8 positive out of 10, respectively) (S1 Table) [48, 49]. All algorithms predict these epitopes to bind with moderate or low affinity to other HLA-class I alleles (B07 and B62, A01, A03, respectively).

For Pf CSP, the maximum predicted IC50 value for a validated 9-mer epitope is 13837.8 nM, corresponding to an extremely low binding affinity for peptide NEEPSDKHI to B44 supertype alleles (representative allele B*40:01). However, IEDB MHC-I binding predicts a binding affinity of 6510.14 nM for the same allele (I_B4001_6510.14). This is also the lowest predicted IC50 for this peptide among the 11 predictions available. In IEDB, this epitope (ID 43604) only contains positive experimental validation assays for binding to mouse H2-Kk alleles and 2 negative MHC binding assays for mouse Mamu-A1*011:01 and human HLA-B*44:02 (S1 Table). Experimental data are extensive for Pf CSP epitope ID 74841 (YLNKIQNSL) including 20 (out of 22) positive assays and show this epitope is immunogenic, binds to HLA-B*07:02, HLA-C*03:03 and mouse H2-b class I alleles, but mostly to the HLA-A*02:01 allele inducing cytotoxicity (chromium-51 cytotoxicity assays), IFN-γ and IL-2 release (ELISA and ELISpot assays) (S1 Table) [50, 51]. This information is well reflected in the HLA-binding prediction data, as this peptide is predicted to bind to alleles from the A02 supertype with an affinity of 17.3 nM, while also showing a strong binding affinity to alleles from the B08 and B62 supertypes (351.1 and 493.8 nM, respectively). Pf CSP epitope ID 42295 (MPNDPNRNV) showed positive results in 14 out of 18 assays performed, and this experimental data shows it binds to HLA-B7 alleles, HLA-B*51:01 and HLA-B*53:01 inducing cytotoxic responses (chromium-51 cytotoxicity assays), and IFN-γ release (ELISpot) (S1 Table) [50, 52]. Accordingly, prediction data indicates strong binding to alleles from the B07 supertype (456.2 nM), whereas the remaining 10 results predict a weak binding affinity to the other allele supertypes.

For Mtb EsxA, the maximum predicted IC50 value for a validated 9-mer epitope is 30302.5 nM, corresponding to a low binding affinity for peptide AWGGSGSEA (ID 189582) to the B62 supertype (representative allele B*15:01). For the same allele, IEDB MHC-I binding predicts a binding affinity of 13739 nM. Experimental data in IEDB shows negative results in 7 assays out of 10 performed, and only 3 positive MHC binding assays (S1 Table) show this epitope binds to HLA-A*30:02 molecules, included in the A01 supertype [53], and the algorithms predict a very low binding affinity of 15981.71 nM (MHC-I binding) and 43641.5 nM (NetMHCpan). On the contrary, experimental data is robust for Mtb EsxA epitope 3064 (AMASTEGNV) with 7/7 positive T-cell and MHC binding assays showing it binds to HLA-A*02:01, HLA-A*30:02, and HLA-A2 alleles and induces IFN-γ release (S1 Table) [53, 54]. This information correlates with prediction data, wherein this peptide is predicted to bind to alleles from the A02 supertype with an affinity of 634.6 nM by NetMHCpan. Additionally, IEDB MHC-I binding predicts this peptide to bind to alleles HLA-A*02:01, HLA-A*02:03, HLA-A*02:06, HLA-A*02:11 and HLA-A*30:02 with predicted affinity of 722.33 nM, 17.19 nM, 1434.02 nM, 11.33 nM and 664.34 nM, respectively. It also has a low binding affinity prediction to the B62 supertype (3423.5 nM). Qualitative binding data shows Mtb EsxA epitope 13195 (ELNNALQNL) binds to HLA-A24 alleles (qualitative binding assays), although ELISpot data shows no induction of IFN-γ release in 2 negative assays (S1 Table) [54, 55]. The best binding prediction for this peptide is low-affinity binding to A26 supertype (4896.2), with predicted affinity to A24 alleles of 29334 nM. Similarly, IEDB MHC-I binding indicates exceptionally low-affinity binding to A24 alleles (28888.98 nM to HLA-A*23:01 and 28049.05 nM to allele HLA-A*24:02).

#### Validated HLA-class II epitopes in final output table (res_classII.csv)

For HCV GP, the maximum predicted IC50 value for a validated 15-mer epitope is 21723.9 nM, corresponding to a low-binding affinity for peptide PPLEGEPGDPDLSDG to allele HLA-DRB3*01:01 (core LEGEPGDPD). This epitope has experimental data indicating it is restricted to HLA-class II alleles and induces IFN-γ release (ELISpot) in the IEDB database albeit with only one positive assay out of 5 (S1 Table) [56]. This peptide has two other prediction results in the final output table–extremely low-affinity binding to alleles HLA-DRB1*07:01 (core LEGEPGDPD, 28796.61 nM), and -DRB1*15:01 (core LEGEPGDPD, 27088.07 nM). HCV GP epitope PLEVIKGGRHLIFCH (ID 48313) has positive experimental MHC binding data in IEDB showing it binds to several HLA-class II alleles (HLA-DR1, -DR11, -DR13, -DR15, -DR3, -DR4, -DR7, and -DRB5) (S1 Table) [57]. In accordance with prediction data, this peptide is predicted to bind strongly or moderately to alleles DRB5*01:01 (core LEVIKGGRH, 122.71 nM), DRB1*07:01 (core IKGGRHLIF, 403.62 nM) and DRB3*01:01 (core IKGGRHLIF, 2860.64 nM). Still, 6 out of 15 total assays (4 T-cell assays and 2 MHC binding) were negative for this 15-mer epitope (S1 Table).

For Pf CSP, the maximum predicted IC50 values for a validated 15-mer epitope is 6994.45 nM, corresponding to a low binding affinity for peptide KPKDELDYANDIEKK to allele DRB5*01:01 (core LDYANDIEK). This epitope (ID 32744) was shown to induce IFN-γ release (ELISpot) and proliferation (^3^H-thymidine proliferation assay) in 2/4 positive assays but no specific allele restriction is known (S1 Table) [58]. Pf CSP epitope 42473 (MRKLAILSVSSFLFV) is a promiscuous epitope with extensive positive experimental data (27 positive assays out of 32) showing it binds to 10 alleles—HLA-DRB1*01:01, -DRB1*04:01, -DRB1*04:05, -DRB1*07:01, -DRB1*09:01, -DRB1*11:01, -DRB1*13:02, -DRB1*15:01, -DRB5*01:01 and -DRB1*12:01 (S1 Table). This epitope induces cellular proliferation (^3^H-thymidine proliferation assay), IFN-γ and IL-10 release (bioassay and ELISA) (S1 Table) [59]. The prediction data for this peptide includes strong binding to alleles HLA-DRB1*07:01 (core AILSVSSFL, 50.78 nM) and -DRB1*15:01 (core ILSVSSFLF, 66.66 nM), and moderately to alleles -DRB3*01:01 (core ILSVSSFLF, 1102.05 nM) and -DRB1*03:01 (core ILSVSSFLF, 1726.82 nM). Yet, one MHC ligand assay was performed for the latter allele with negative results (S1 Table) [60].

For Mtb EsxA, the maximum predicted IC50 value for a validated 15-mer epitope is 10209.32 nM, corresponding to a very low binding affinity for peptide WGGSGSEAYQGVQQK (core EAYQGVQQK) to allele HLA-DRB5*01:01. This example illustrates a disagreement between predictors, as IEDB MHC-II binding predicts this full peptide/core combination binds to DRB5*01:01 allele with an affinity of 1170.1 nM. Furthermore, evidence in the IEDB database showed this epitope (ID 226404) induces IFN-γ release (ELISpot) and is restricted to HLA-DRB1 alleles but with only one positive assay (S1 Table) [61]. The prediction algorithms again disagree on full_peptide/core combinations binding to DRB1 alleles. IEDB MHC-II binding predicts the combination WGGSGSEAYQGVQQK/WGGSGSEAY binds with extremely low affinity to alleles DRB1*03:01 (33097.7 nM) and DRB1*15:01 (15465.1 nM). NetMHCIIpan has no predictions for DBR1 alleles for the full_peptide/core combination WGGSGSEAYQGVQQK/WGGSGSEAY, and the two prediction algorithms do not agree on the peptide core EAYQGVQQK binding to DRB1 alleles (only NetMHCIIpan has low binding affinity predictions for allele DRB1*03:01, 20821.11 nM). Therefore, there are no full_peptide/core combinations yielding IC50 predictions for this peptide and DRB1 alleles. By contrast, both algorithms predict Mtb EsxA epitope QGNVTSIHSLLDEGK (core VTSIHSLLD) to bind to DRB1*07:01 (804.18 nM), and with lower affinity to DRB5*01:01 and DRB1*03:01 (2493.15 nM and 6448.37 nM, respectively). IEDB MHC-II binding predicts stronger binding affinities for this full_peptide/core combination for the same alleles (59.5 nM, 74.1 nM and 2505.9 nM, respectively); binding to other alleles are not predicted by this algorithm. Qualitative binding information in IEDB shows epitope QGNVTSIHSLLDEGK (ID 161673) is strongly associated with allele DRB1*04:05, with the same core prediction (VTSIHSLLD), and weak associations with DRB1*1501 and DRB1*1502 alleles, with 4/4 positive assays (S1 Table) [61]. Only NetMHCIIpan predicts this full_peptide/core combination to bind to DRB1*1501 (1127.94 nM). Additionally, the full_peptide/core combination QGNVTSIHSLLDEGK/IHSLLDEGK is only predicted to bind to allele DRB4*01:01 (498.3 nM) by the IEDB MHC-II binding predictor. NetMHCIIpan predicts strong binding to this allele with a different core (VTSIHSLLD, 578.09 nM).

### Sensitivity and specificity of different filtering options

By applying some filtering criteria to the final output tables, one can significantly reduce the epitope lists to test, while attempting to increase the chances of selecting and not rejecting immunogenic epitopes. Some example filters were applied based on the information retrieved from the validated epitopes in IEDB (mainly maximum/minimum predicted IC50, promiscuity, mismatches, hydrophobicity). The final criteria to define the best filters were the highest list reduction with the best values for sensitivity/specificity (minimum 60%, if possible).

**Fig 3:**
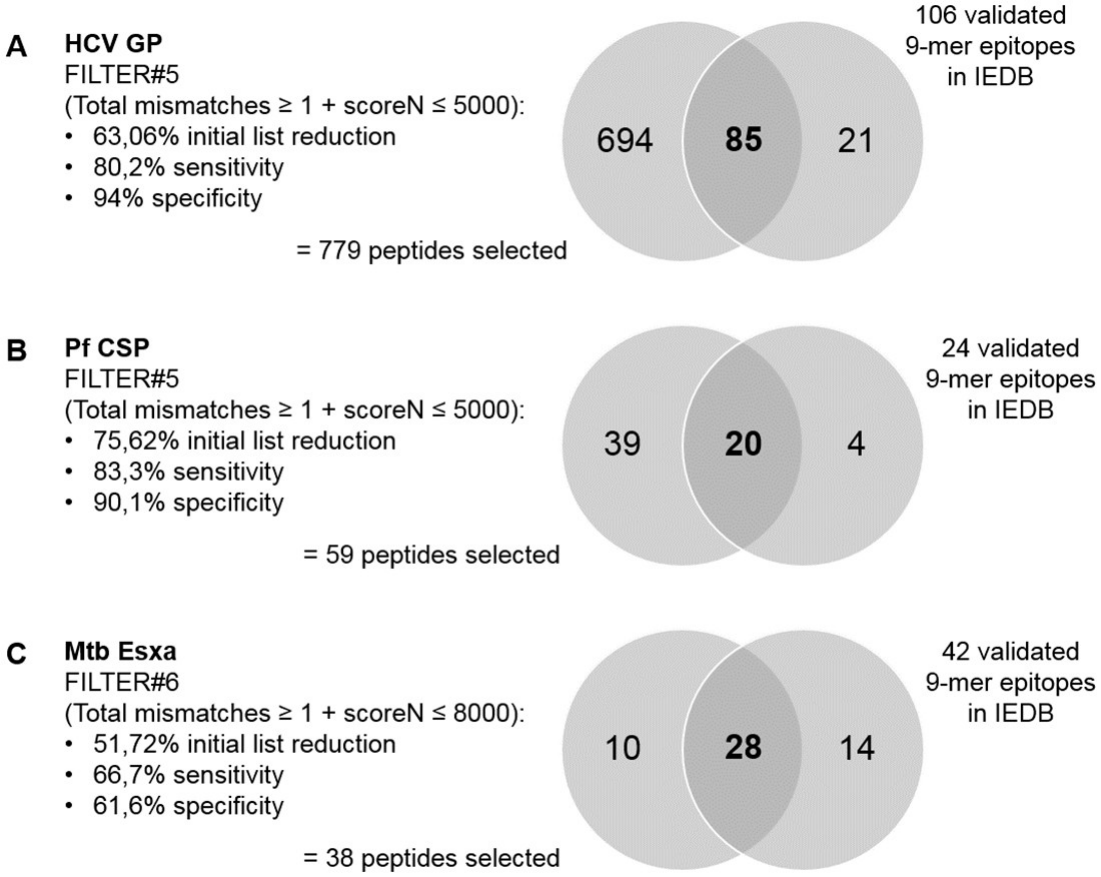
Sensitivity and specificity of example filters applied to final HLA-class I results table from SILVI.

The example low stringency filters applied on HLA-class I results on total mismatches and predicted IC50 values by NetMHCpan allowed to reduce the initial peptide list by 52–76%, while retaining sensitivity of 67–83% and a specificity from 62–94% (Fig 3). Other example filters yielded good results for Pf CSP and HCV GP proteins, namely, FILTER#3 (total mismatches ≥ 1 and scoreN ≤ 1000) which resulted in a list reduction of 88,4% and 80,3%, respectively, with a sensitivity of 62,5 and 63,2% and specificity of 93,4% and 96,2%, respectively (S2 Table).

Applying FILTER#5 (total mismatches ≥ 1 + scoreN ≤ 5000 nM) to the final HLA-class I prediction results table for the HPV proteome, we obtained a 65,7% reduction of the initial peptide list (758 peptides selected, with 1287 predictions).

**Fig 4:**
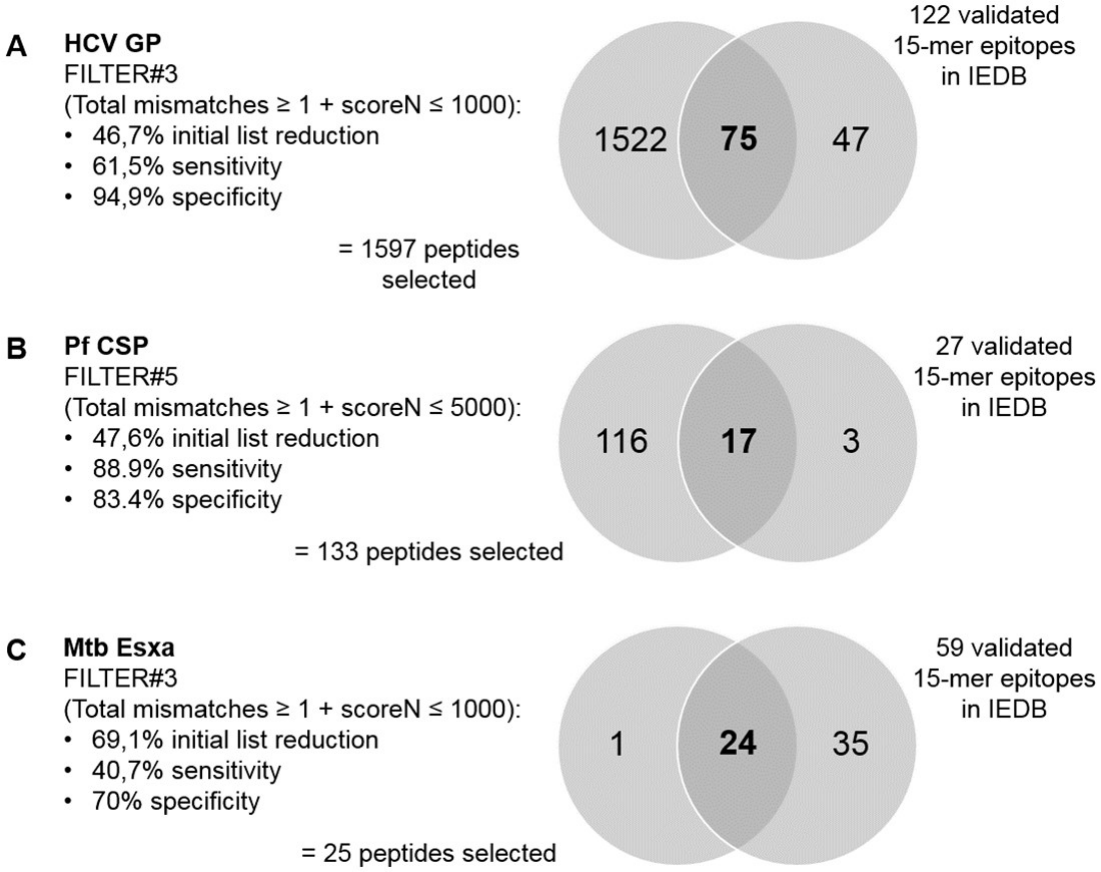
Sensitivity and specificity of example filters applied to final HLA-class II results table from SILVI.

The example low stringency filters applied on HLA-class II results on total mismatches and predicted IC50 values by NetMHCpan allowed to reduce the initial peptide list by 47–69%, while retaining sensitivity of 41–85% and a specificity of 70–95% (Fig 4 and S3 Table).

Applying FILTER#3 (total mismatches ≥ 1 + scoreN ≤ 1000 nM) to the final HLA-class II prediction results table for the HPV proteome we obtained a 39,2% reduction of the initial peptide list (1342 peptides selected, with 2995 predictions).

## Discussion

The ability to compare different data sources and to synergistically combine various algorithms in epitope prediction remains challenging. The potential of the datasets generated by omics technologies is limited by the lack of appropriate computer-based tools to manage and integrate the vast amount of epitope prediction data. Experimental validation steps restrict the number of peptide candidates to test, meaning that these candidates must be carefully selected to increase the chances of selecting immunogenic peptides. The successful identification of immunogenic epitopes depends on both the quality of used immunoinformatic algorithms and on the rationale behind epitope selection criteria. These criteria must be adjusted to the biological question at hand; they should be permissive enough not to reject immunogenic epitopes falsely, and sufficiently restrictive to filter HLA-binding prediction data effectively.

SILVI (short for: from *in silico* to *in vivo*) is a workflow written in R language that was developed to assist the selection of epitopes predicted by state-of-the-art HLA-binding affinity algorithms using also other sequence-specific features such as conservation among pathogenic species and homology to host. To help researchers make use of SILVI, the README file (available at github.com/JoanaPissarra/SILVI2020 and https://doi.org/10.5281/zenodo.6865909) includes in-depth explanations on the necessary downloads and dependencies, input file preparation and script utilisation. Researchers are free to adapt the script to particular conditions and interests.

The SILVI pipeline was adapted to integrate data from the most widely used epitope prediction algorithms with high accuracy HLA-binding predictions [62], and homology alignments [45]. Based on this information, together with conservation among species, physical-chemical properties, and target population HLA restriction, this epitope selection pipeline presents all results in a single table, manageable in a spreadsheet analysis software. Users can then filter the data and reduce the number of peptides to test while increasing the chances of identifying immunogenic peptides. SILVI was developed for human T-cell epitope selection, so only human alleles are expected in this version. Its extension for epitopes of interest in veterinary immunology is desirable yet requires further implementation.

To generate the input data, users should choose their protein sequences according to selected features such as abundance, expression and subcellular localization. SILVI will not apply these filters; it is up to the user to supply high-quality data from the best antigen pool. We recommend the use of databases such as EUpathDB [63] or UniProtKB [64] and transcriptomic and proteomic information to design the initial protein candidate list and integrate gene expression data for the protein annotation from the start. Once the protein antigen pool is selected, protein sequences in fasta format are retrieved and directly uploaded in the online servers to perform HLA-binding predictions. SILVI allows users to perform an epitope-based selection, aiding the diversification of antigen sources. SILVI is designed to analyse several species-specific sequences per protein antigen (1 *seq_num* = 1 species-specific sequence). In the first selection step (step A), SILVI uses homology as a positive selection filter for highly conserved epitopes among pathogenic species (each protein can include several species-specific sequences in fasta format). SILVI strictly considers identical epitope sequences from several species-specific protein sequences (*common_among_seq_nums* filter), so it is a suitable tool for well-conserved proteins or the discovery of highly conserved epitopes. Nevertheless, users may run all protein sequences in a single file (1 *seq_num* = 1 protein, regardless of strain or species), i.e. example#4 (HPV proteome). In this case, SILVI will return all potential epitopes from any given protein ignoring conservation among species-specific sequences. The *common_among_predictors* filter concedes a higher level of confidence in predicted immunogenicity, since non-redundant prediction algorithms independently predict a given epitope to be a strong binder [27, 34]. These two filters are the only rejection steps included in SILVI. All subsequent steps add information without filtering out peptides. Also, even if the same linear sequence is predicted as an epitope, many will not be assigned the same HLA restriction and in this case “promiscuity” will equal 0. This reveals the limitations of available algorithms and the importance of using combinatorial approaches for epitope prediction. SILVI will accept virtually all available algorithms so long as the input data is correctly formatted.

For HLA-class II binding predictions, the total epitope size is 15-mer, which includes the 9-mer motif for HLA binding: the epitope core or register. The algorithms’ predictive power correlates better with the core prediction [29]. By comparing core predictions, we compare predictors and consider all potential cores within a 15-mer peptide, selecting the best full_peptide/core combination according to predicted IC50.

In selection steps B and C, homology to host proteins (information from BLASTp) is added for epitope ranking and description [28]. HLA-class I alleles have known anchor binding positions, wherein 2 or 3 amino-acids are essential for stable peptide-MHC interactions [14]. Through total and anchor position mismatch counts it is possible to detect epitopes that show high homology with human proteins, thereby reducing the chances of unwanted cross-reactivity and autoimmune responses [28].

The example proteins demonstrate the ability to integrate relevant HLA-binding predictions from different algorithms and extra information to help epitope selection into a single output table. The example proteins include several experimentally validated epitopes, some of which with a high number of positive assays in the IEDB. Since SILVI currently only accepts 9-mer and 15-mer epitopes, we selected the total 9-mer and 15-mer validated epitopes from the IEDB database, which we used to analyse the frequency distribution of the several properties included in SILVI’s output table (molecular weight, hydrophobicity, isoelectric point, total mismatches, promiscuity, HLA restriction, binding affinity, and anchor mismatches to HLA-class I predictions). While with a highly variable number of total assays and positivity rates in the IEDB, most validated epitopes have corresponding favourable prediction data. In contrast, others are predicted to be non-binders by the algorithms, disagreeing with experimental data in the IEDB database; this again highlights limitations of such HLA-binding prediction algorithms in epitope prediction.

Overall, there is a high concordance between IEDB and NetMHCpan (promiscuity ≥ 1). Additionally, IC50 predictions are reliable, depending on allele restriction and the general representation of pathogens in the algorithms’ databases, providing a useful filter to manage epitope prediction data. Still, as some validated epitopes with experimental evidence in the IEDB for cytotoxicity are predicted to have very high IC50 values, it illustrates current algorithms may fail to predict binding affinity and/or immunogenicity, so low stringency filters should be used.

The filters applied to SILVI’s output allowed a pronounced reduction of the initial epitope lists. Despite this broad selection, and particularly for HCV GP and HPV proteome, too many peptides are selected, which may be an issue for the experimental validation at reasonable cost. Users may also select particular epitope-rich regions; make relative comparisons according to the predicted HLA restriction to choose the best value per supertype or allele, or perform additional epitope analysis subsequently (e.g. immunogenicity, clustering, or population coverage analysis).

Binding affinity is a crucial characteristic of peptide immunogenicity and the general cut-off value of 500 nM has been extensively used in T-cell epitope selection. Yet, validated epitopes in the IEDB have divergent predicted IC50 values, ranging from predicted strong binders to non-binders, particularly the Pf CSP and Mtb EsxA examples. For HCV GP and Pf CSP over half of validated epitopes have predicted binding affinities below 1100 nM, which hints on a potential less stringent IC50 cutoff value. We use low stringency IC50 cut-off values in the examples, but SILVI allows the use of broad filters and/or allele-specific filters, which are more predictive [13]. Users may also analyse the final output table applying allele-specific binding information to establish cut-off values and promiscuity, which is also a common characteristic observed for validated epitopes. BLASTp information on homology to host proteins from validated epitopes also validates the use of mismatches with human proteins as a filtering criterium, as at least one total mismatch is found and allowed to reduce substantially the epitope lists.

In contrast to HLA-class I, the total number of HLA-class II predictions are generally more extensive, which correlates with the prediction algorithms’ performance, higher epitope promiscuity, and the existence of multiple binding cores within a peptide, which can be used as additional filters.

No single universal filter will be suitable for all protein antigens or T-cell epitope selection workflows. Some experimentally validated epitopes present ambiguous experimental data and low HLA-binding predictions, revealing current limitations. Users may also add more data from other immunoinformatic tools by integrating those results into the SILVI dataframe.

Present limitations of this pipeline version are: epitope size restricted to 9-mer and 15-mer peptides, which correspond to only around a quarter of total validated epitopes; user-dependent preparation of .csv files with HLA-binding prediction data; and adaptations needed to adjust the *common_among_seq_nums* filter to analyse multiple proteins without species-specific sequences. However, as SILVI is written in the R language, a free software environment widely used by researchers across different research fields, all existent included features and criteria can be modified. For instance, including an automated BLASTp analysis to reduce user-dependent steps. More importantly, SILVI is entirely open-access. Also, any script improvements will be made in a community-driven manner, tackling different scientific challenges, and paving the way for broader discussions on immunogenicity predictions and epitope selection, which can eventually lead to a packaged version of the SILVI pipeline or the development of a graphical user interface (GUI). Furthermore, continuous improvement of data analysis *in silico* tools like SILVI will ultimately decrease the need for conventional animal testing, reduce the time needed for pre-clinical development, and fast-track product deployment.

## Conclusions

SILVI uses available high-performing HLA-binding predictors and relevant rational criteria associated with immunogenicity, allowing a swift selection of T-cell epitopes from large datasets and thereby restricting the total number of peptides to test at the bench while increasing the chances of selecting the most conserved and immunogenic epitopes. This pipeline also helps epitope-mapping experiments by identifying *in silico* high immunogenicity regions in several antigen candidates. The integration of BLASTp data is a great advantage since it is the most well-established method to find homologous sequences in all host organisms and provides excellent insight regarding the cross-reactive potential of an epitope. SILVI applies to any pathogenic organism, allele restriction and prediction algorithm. It allows users to integrate diverse outputs with the freedom to select the most relevant criteria in a fast and reproducible manner. Finally, SILVI is customisable allowing for additional development (automated BLASTp, refined and/or extra criteria, synthetic summary, etc). We believe that this open-source tool will significantly help future epitope-based vaccines and immunotherapies design.

## Materials and methods

### Script development

**Fig 5:**
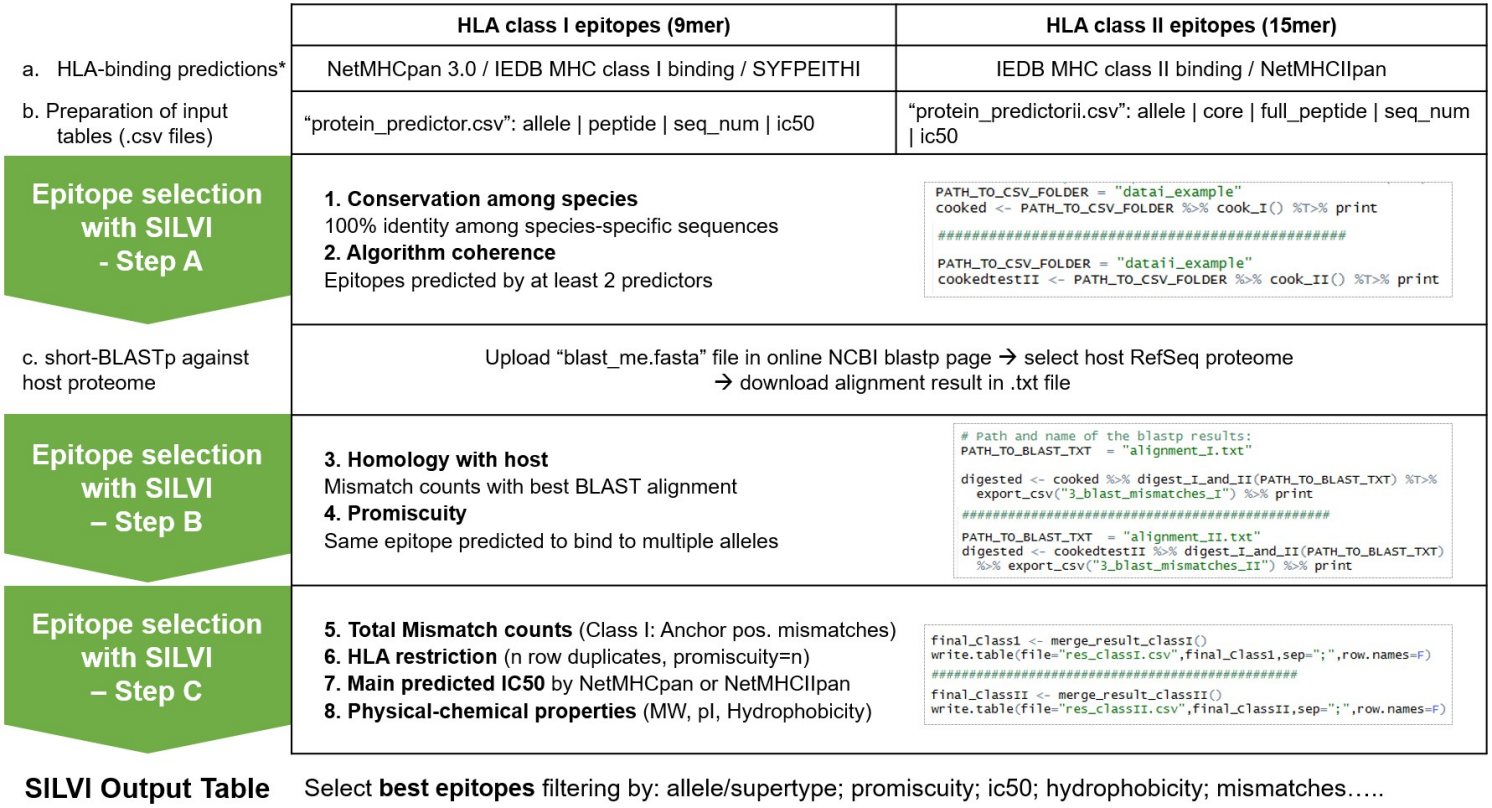
SILVI’s computer-based pipeline for T-cell epitope selection. Steps in black (a. to c.) are user-dependent; steps in green (“Epitope selection with SILVI–Step A to C”) are automatically applied by the script. *Users input protein sequences in web-based algorithm interfaces and export results.

SILVI’s workflow involves two user-dependent steps (input HLA-binding prediction data and BLASTp alignment results against the target proteome) and three semi-automated steps (Fig 5), detailed in the file README.md. SILVI is an open-source script, written in the R programming language (https://www.r-project.org/) and is freely available to download from GitHub and Zenodo (see github.com/JoanaPissarra/SILVI2020 or https://doi.org/10.5281/zenodo.6865909 for instructions). The R package dependencies are tidyverse [65]; stringr [66]; and Peptides [67].

#### Input data

Input data consists of 9- or 15-mer predicted epitopes and respective predicted binding affinity to several HLA alleles, according to the target population (allele list upload files for world population coverage are available), generated by HLA-binding prediction algorithms. For HLA-class I prediction data, users must prepare the .csv files containing results from at least two non-redundant and well-performing HLA class-I binding prediction algorithms (e.g. NetMHCpan 4.0 [68], IEDB MHC-I binding [69], and SYFPEITHI [70]). A low stringency score filter can be applied to select the top percentile of predicted epitopes (e.g. 10–50%) to reduce initial epitope lists. HLA-class I peptide.csv files should be named “proteincode_predictorcode.csv” and should contain the columns: “allele”; “peptide” (9-mer); “ic50” or “score”; “seq_num” (sequence_number).

For HLA-class II prediction data, users must prepare .csv files containing results from at least two non-redundant and well-performing HLA class-II binding prediction algorithms (e.g. NetMHCIIpan [71], IEDB MHC-II binding [30, 33]), with the desired cut-off value (e.g. top 10–50%). HLA-class II peptide.csv files should be named “proteincode_predictorcodeii.csv” and contain the columns: “allele”; “full_peptide”; “core”; “seq_num”; “ic50”; “rank” (Fig 1a and 1b).

Users may check the file code/db_headers to confirm the correct column names, for both HLA-class I and class II predictions.

#### First selection step (step A)

The script imports and integrates all data into a single data frame class in R and, to address conservation among pathogenic species, by default, it directly compares all 9-mer peptides or cores among different sequences from the same protein, selecting only the ones that are 100% identical (*common_among_seq_nums* filter). Simultaneously, the script compares the full epitope lists generated by each prediction algorithm used and selects only the 9-mer epitopes predicted by at least two predictors to bind to a given allele or supertype (*common_among_predictors* filter). Predicted HLA restriction is added to the exported table (Fig 5 - Epitope selection with SILVI- step A).

SILVI’s step A generates a single data frame where all input information is gathered and applies the only truly selective filters in the script (*common_among_seq_nums* & *common_among_predictors*). SILVI’s steps B and C simply add extra information without decreasing the list size, to allow the user to characterize the predicted epitopes and perform tailored selections.

HLA-class I epitope selection: users open the file ‘Fire_classI.R’, introduce the directory pathway to input .csv files and run the first code lines (step A). To assign HLA restriction, the comparison is made per supertype (11 supertypes), which allows the comparison among predictors [72]. The correspondences between allele and supertypes (11) are in the file /code/map_supertype_alleles.csv, where users may add new alleles.

HLA-class II epitope selection: users open the file ‘Fire_classII.R’, introduce the directory pathway to input .csv files and run the first code lines (step A). To assign HLA restriction, SILVI compares per allele, so it is important to perform predictions with the same allele lists.

As an intermediary output, the script generates a .csv file for the initial epitope list (“1_common_I/II.csv”) with all collected information thus far (source protein, all predicted allele/supertype restrictions, peptide sequence, number of predictors, number of seq_nums, scores and raw data file). Step A also generates a .txt file with all 9-mer peptides or cores in FASTA format, to be uploaded for online short-BLASTp analysis (“1_blast_me.fasta”). Users choose the host reference dataset (e.g. *Homo sapiens* taxid: 9606, RefSeq) and desired alignment parameters (e.g. default for short sequences). We recommend users to reduce the size of the alignment result file by selecting a maximum of 10 aligned sequences to display, in the ‘Max target sequences’ option in algorithm parameters, general parameters tab. Users download the short-BLASTp alignment result in .txt file format to be imported again in R (Fig 5c).

#### Second step (B)

Users introduce the path and name of the short-BLASTp result file and run the code (step B). To verify the alignment result file is complete, SILVI confirms all peptides were included in the alignment result file. In this step, SILVI reads the first short-BLASTp alignment hit for each 9-mer epitope or core and counts the position-specific matches (“m_1” to “m_9”). Positive residues are considered a match, and when alignment gaps are introduced the succeeding positions are considered as mismatches (Fig 5 - Epitope selection with SILVI- step B). SILVI calculates the total number of matches/mismatches in each epitope or core (“match” and “mismatch”), as well as supertype-specific anchor position mismatches for class I peptides (“anchormm”). SILVI generates two intermediary output .csv files (“2_common_blast_I/II.csv” and “3_common_blast_I/II.csv”).

#### Third step (C)

Once the new dataframe is generated by the 2^nd^ step, users run the last code lines and SILVI adds extra information: molecular weight, hydrophobicity using the GRAVY (grand average of hydropathy) hydrophobicity index, calculated by adding the hydropathy value for each residue and dividing by the length of the sequence [73], and pI using the Bjellqvist pK scale with the Peptides package [67]. Moreover, SILVI calculates the “promiscuity”, the total number of alleles/supertypes to which a given epitope is predicted to bind to, and duplicates the rows according to this information, allowing selections based on predicted HLA restriction. Also, SILVI highlights the predicted IC50 to specific alleles from the NetMHCpan algorithm (“scoreN”) to help the user select the top predicted binders (Fig 5 - Epitope selection with SILVI- step C). All IC50 predictions are kept in the “score” column.

As the final output, SILVI generates a .csv file (“res_classI/II.csv”) containing the initial peptide list from the first selection step, plus the short-BLASTp alignment results (step B) and all the other relevant information added in the last selection step (step C). The user is then free to analyse the list, complement with more data if needed, and prioritize the different criteria as desired.

### Script implementation

#### Example proteins

The IEDB database includes thousands of validated pathogen-specific epitopes, and the following search strategy was used to find extensively validated T-cell epitopes: Linear epitopes + Positive assays only + No B cell assays + Human host + Infectious Disease (S1 Table). The Hepatitis C Virus (HCV) genome polyprotein (GP), *Plasmodium falciparum*’s circumsporozoite protein (CSP), *Mycobacterium tuberculosis*’s 6 kDa early secretory antigenic target and the HPV proteome were used as example proteins to undergo T-cell epitope selection with SILVI.

HLA-class I binding predictions were performed on: 1) NetMHCpan 4.0 [68] predictions for 11 supertype representative alleles, 2) IEDB MHC-I binding [69] (Prediction Method Version 2013-02-22, recommended predictions for 36 alleles), and 3) SYFPEITHI predictions (default predictions for 22 alleles). The allele reference panel provided by IEDB MHC-I binding (27 alleles) was expanded to include 36 alleles (S4 Table). Individual csv files were prepared with all predicted epitopes and named “protein_predictori.csv”.

HLA-class II binding predictions were performed on: 1) NetMHCIIpan [71] (predictions for 7 alleles), and 2) IEDB MHC-II binding [33] (recommended predictions for 7 alleles), and *nn_align* core and IC50 values [30]. The allele reference panel of 7 alleles was selected as suggested by IEDB MHC-II binding and described in Paul et al, 2015 [74] (S5 Table). Individual csv files were prepared with all predicted epitopes and named “protein_predictorii.csv”.

## Supporting information

S1 Fig Distribution analysis of class-I epitope properties. Validated HLA-class I 9-mer epitopes from the IEDB database were characterized according to hydrophobicity (A), isoelectric point (B), molecular weight (C), total mismatches (D), anchor mismatches (E), HLA restriction (F), minimum binding affinity prediction (G), and all predicted binding affinities (H).
(TIF)

S2 Fig Distribution analysis of class-II epitope properties. Validated HLA-class II 15-mer epitopes from the IEDB database were characterized according to hydrophobicity (A), isoelectric point (B), molecular weight (C), total mismatches (D), promiscuity (E), HLA restriction (F), minimum binding affinity prediction (G), and all predicted binding affinities (H).
(TIF)

S1 Table Validated epitopes from IEDB.org and corresponding experimental data (T-cell and MHC binding assays). (XLSX)

S2 Table Sensitivity and specificity of example filters applied on SILVI HLA-class I results table. (XLSX)

S3 Table Sensitivity and specificity of example filters applied on SILVI HLA-class II results table. (XLSX)

S4 Table HLA-class I alleles and supertypes. Supertypes according to Sydney J. et al 2008 BMC Immunology 9:1.
(XLSX)

S5 Table HLA-class II alleles. IEDB 7 allele reference set according to Paul S et al 2015 Journal of Immunological Methods 422:28–34.
(XLSX)
