# Supplementary figures and images for: SILVI, an open-source pipeline for T-cell epitope selection
Source: PLoS One. 2022 Sep 7;17(9):e0273494. doi: 10.1371/journal.pone.0273494 (PMC9451077; doi:10.1371/journal.pone.0273494)

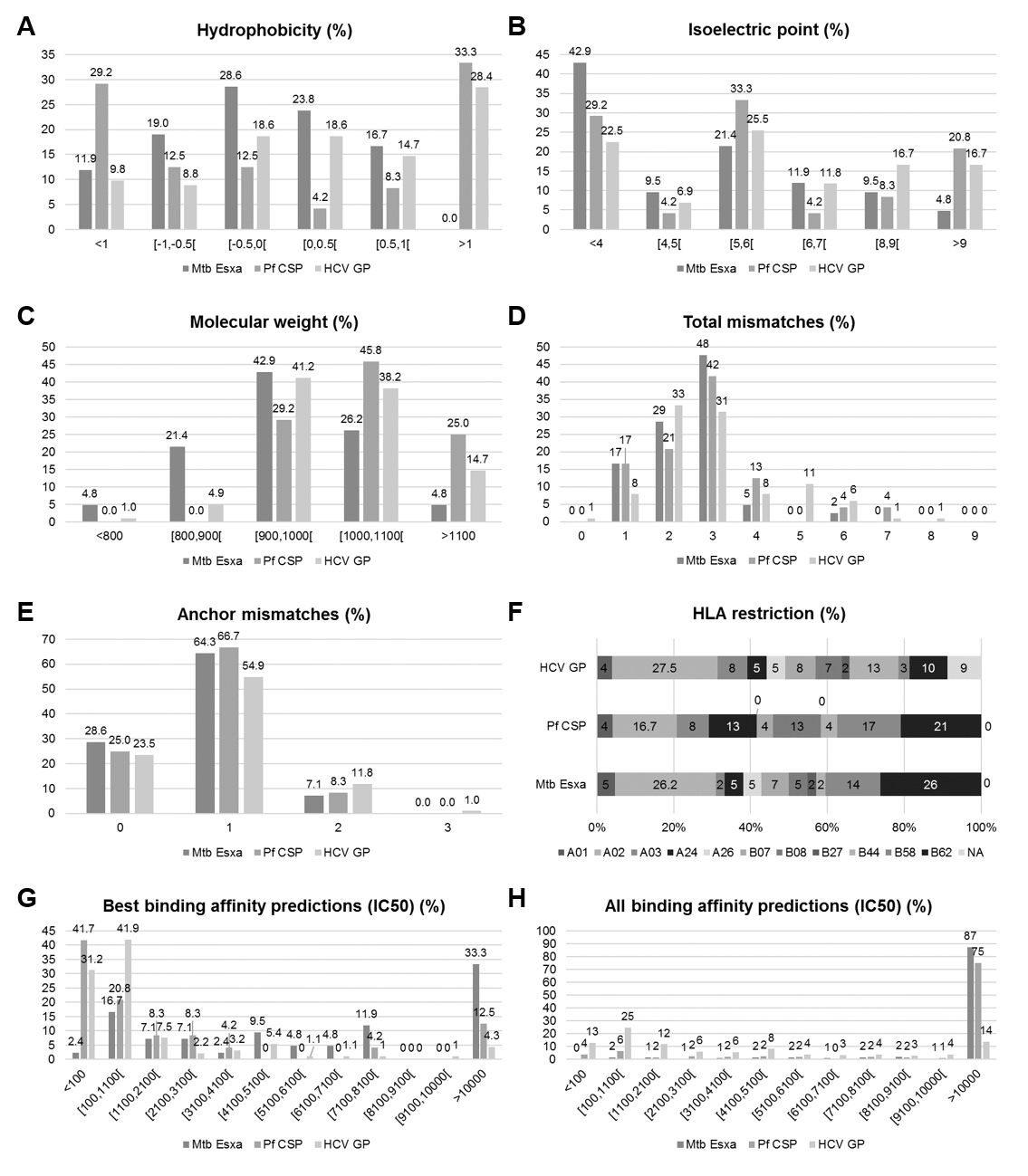

Supplement: S1 Fig — Validated HLA-class I 9-mer epitopes from the IEDB database were characterized according to hydrophobicity (A), isoelectric point (B), molecular weight (C), total mismatches (D), anchor mismatches (E), HLA restriction (F), minimum binding affinity prediction (G), and all predicted binding affinities (H). (TIF) [file pone.0273494.s001.tif]

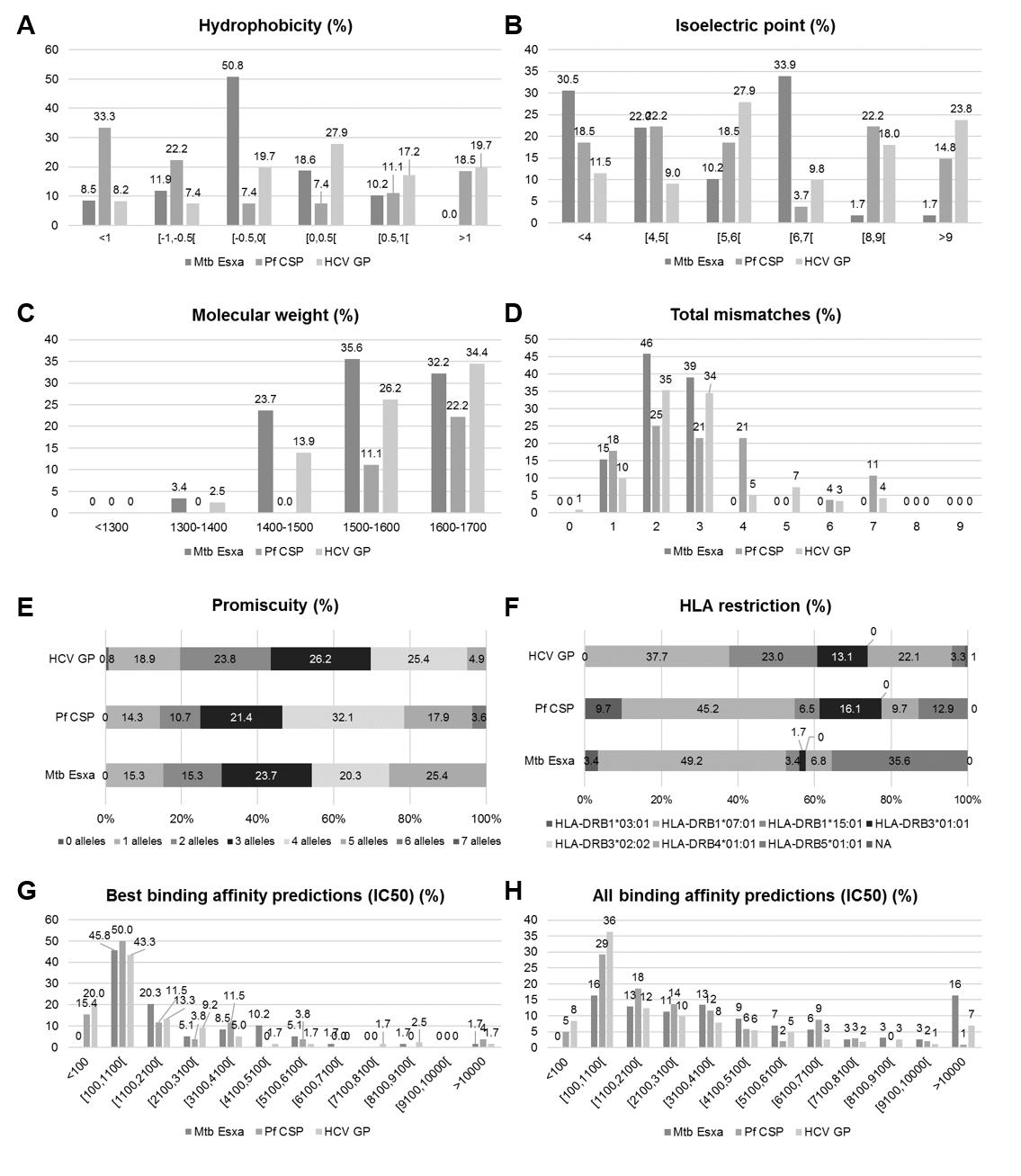

Supplement: S2 Fig — Validated HLA-class II 15-mer epitopes from the IEDB database were characterized according to hydrophobicity (A), isoelectric point (B), molecular weight (C), total mismatches (D), promiscuity (E), HLA restriction (F), minimum binding affinity prediction (G), and all predicted binding affinities (H). (TIF) [file pone.0273494.s002.tif]
